# Anti-Hypertensive Activity of Some Selected Unani Formulations: An Evidence-Based Approach for Verification of Traditional Unani Claims Using LC-MS/MS for the Evaluation of Clinically Relevant Blood Parameters in Laboratory Rats

**DOI:** 10.3390/jcm11154628

**Published:** 2022-08-08

**Authors:** Md. Adil Shaharyar, Rudranil Bhowmik, Obaid Afzal, Abdulmalik S. A. Altamimi, Sami I. Alzarea, Waleed Hassan Almalki, Sk Zeeshan Ali, Pallab Mandal, Avishek Mandal, Mohd Ayoob, Imran Kazmi, Sanmoy Karmakar

**Affiliations:** 1 Bioequivalence Study Centre, Department of Pharmaceutical Technology, Jadavpur University, Kolkata 700032, West Bengal, India; 2 Department of Pharmaceutical Chemistry, College of Pharmacy, Prince Sattam Bin Abdulaziz University, Al-Kharj 11942, Riyadh, Saudi Arabia; 3 Department of Pharmacology, College of Pharmacy, Jouf University, Sakaka 72341, Al-Jouf, Saudi Arabia; 4 Department of Pharmacology, College of Pharmacy, Umm Al-Qura University, Makkah 21955, Makkah, Saudi Arabia; 5 The Calcutta Unani Medical College and Hospital, 8/1, Abdul Halim Lane, Kolkata 700016, West Bengal, India; 6 Department of Biochemistry, Faculty of Science, King Abdulaziz University, Jeddah 21589, Makkah, Saudi Arabia

**Keywords:** systolic blood pressure, unani system, adrenaline, ECG, noradrenaline

## Abstract

**Background:** Systemic arterial hypertension, which is associated with an increased risk of cardiovascular disease(CVD), is the most significant modifiable risk factor for mortality and morbidity worldwide. WHO has recognized Unanipathy as an alternate system of medicine. The aim of the present study is to investigate the anti-hypertensive activity of some selected unani formulations using L-NAME model. **Method:** Group I or hypertensive control group: L-NAME administered for 7 days and left for the next 7 days; Group II or KASgroup: L-NAME administered (i.p) for 7 days and L-NAME + KAS (1000 mg/kg b.w) for the next 7 days; Group III or DMM group: L-NAME administered (i.p) for 7 days and L-NAME + DMM (2000 mg/kg b.w) for the next 7 days; Group IV or MSR group: L-NAME administered (i.p) for 7 days and L-NAME + MSR (300 mg/kg b.w) for the next 7 days; Group V or HJ group: L-NAME administered (i.p) for 7 days and L-NAME + HJ (113 mg/kg b.w) for the next 7 days; Group VI or KGS group: L-NAME administered (i.p) for 7 days and L-NAME +KGS (2000 mg/kg b.w) for the next 7 days. Non-invasive systolic blood pressure and RR-interval (ECG) was measured. Plasma was investigated forsodium, potassium, nitrite, ANP, adrenaline, noradrenaline and aldosterone on day 0, 7 and 14 using LC-MS/MS. **Result:** Treatment showed a non-significant lowreduction in SBP (systolic blood pressure) of KAS, MSR and HJ while that of DMM was quite significant (*p* < 0.05), but in the case of KGS, SBP increased. DMM on day 14 significantly (*p* < 0.05) reduced plasma nitrite while no significant plasma Na+ was noted. In the case of both DMM and KGS, potassium increased significantly (*p* < 0.05) on day 14. No significant changes in plasma ANP and aldosterone was observed against DMM and KGS while blood levels of adrenaline and noradrenaline significantly (*p* < 0.05) changed. No significant change in body weight was found. **Conclusions:** L-NAME KAS, MSR and HJ showed no change in SBP while DMM showed a significant reduction in SBP with decreased plasma nitrite. Probably, DMM may have anti-hypertensive activity mediated through NO inhibition while KGS may involve central sympathomimetic action.

## 1. Introduction

Among the many serious medical conditions, hypertension is one where there is an increased risk for the heart, brain, kidney and other organs. Hypertension plays a pivotal role behind CVD mortality and disease burden worldwide. One of the World Health Organization’s global non-communicable disease (NCD) main goals, adopted in 2013, was to reduce the occurrence of hypertension to 25% by 2025 compared to 2010 [1]. Over the last decade, the combination therapies constituting multiple biologically active components have replaced the single component-based therapies. Considering the insufficiency of the single drug anti-hypertensive treatment in chronic conditions, a multiple drug regimen, addressing different anti-hypertensive targets at the same time, has become increasingly popular for clinicians [2].

The World Health Organization (WHO) has recognized the Unani system of medicine as a form of alternative medicine used by people globally.It considers a person’s entire body, mind and spirit. It sees the human body as one cohesive system made up of four basic parts with four different temperaments. The physical qualities and inherent disposition of a person are reflected in their temperament. The human body is susceptible to several ailments due to temperamental imbalances [3]. Contemplation of unani literature points out that some of the formulations have been used for centuries in the management of cardiovascular diseases. In the Unani system of medicine, polyherbal formulations are generally named on the basis of the chief ingredient present (i.e., the ingredient which is considered to be mainly responsible for preparation’s purpose—OR—sometimes the component present in the maximum quantity). An example is Habb-e-jadwar (HJ), where jadwar (5.136 mg per 240 mg of a pill) or Delphinium denudatum Wall. root is the main component. Similarly, Khamira Abreesham Sada (KAS), containing Abreesham (2.193 mg per g of KAS) or Bombyx mori cocoon as the main component. A total of 27.75 mg of *Rosa damascene* Herrm. is present per g of Mufarreh Shaikh ur Rais. Khamira Gawzaban Sada contains “Gawzaban”, i.e., *Borago officinalis* L., which is the main component. As per the label claim of this commercial unani product, 17.544 mg of Borago officinalis L. (leaf and flower) per g weight of the KGS and 26.4 mg of Berberis aristata DC (fruit) per g weight of the DMM arepresent as the chief components in their respective formulations [4,5]. The complete composition of these formulations are mentioned in Table 1, Table 2, Table 3, Table 4 and Table 5 [4,5,6]. Khamira Gozaban Sada (KGS)and Khamira Abreesham Sada (KAS) have been reported to be used exclusively as Muqavvi Qalb (cardiac tonic) and Khafqan (palpitations) [4,5] while Dawaul Misk Motadil (DMM) has been reported to be used as Muqavvi Qalb (cardiac tonic),Muqavvi Jiger (liver tonic) and particularly in Khafqan Saudavi (melancholic palpitations) [5]. Mufarreh Shaikhurais has been used as Mufarreh Qalb (refrigerant), Dafe Khafqan (relieves palpitations) and Muqavvi Qalb (cardiac tonic) [5]. Similarly, Habb-e-Jadwar (HJ) has been useful as Zofe Bah (sexual weakness), Jiryan (spermatorrhea), Sual (cough) and Nazla Muzmin (chronic cold) [5]. These formulations are frequently prescribed in almost all of the Unani hospitals in and around India. In India, the Unani system of medicine has been in practice since 1350 AD. At present this medical practice is still in use and is believed to be effective with minimal adverse events. Very few scientific studies are actually available on these polyherbal preparations. Our study was to contribute scientific input in the pursuit of creating scientific literature about this particular age-old system of medical practice. We have collected information from the Unani practitioners who still believe in the efficacy of this system of therapy, where efficacy may vary but adverse events are infinitesimally insignificant [7].

The background of this study stems from the frequent prescription of these formulations by Unani physicians to patients suffering from hypertension alone or hypertension accompanied by other cardiovascular complications. The present pharmacological investigation aims to verify whether these traditional formulations, which are currently in clinical practice in and around India, possess any anti-hypertensive activity, particularly against L-NAME (N^ω^-nitro-L-arginine methyl ester)-mediated experimental hypertension. 

## 2. Materials and Methods

### 2.1. Chemicals

The L-NAME C_7_H_15_N_5_O_4_.HCl (Sigma Aldrich, Burlington, MA, USA, catalogue no: N5751) was purchased from Sigma Aldrich. The KGS(batch no: MKHO35A); KAS (batch no. 0KM0109); MSR (batch no. 0K00010); DMM (batch no. MDE019) and HJ (batch no.0KN0001) (Hamdard Laboratories, Mfg Lic. No. U-212/78/2014) were a kind donation from The Calcutta Unani Medical College and Hospital, 8/1,Abdul Halim Lane, Kolkata, West Bengal 700,016. The Na^+^, K^+^ and the Cl^−^ ELYTE-3 estimation kit was provided by Coral Clinical Systems; catalogue no: SOD/POT/CL (ELYTE-3)/15T/CR. The Griess Reagent (catalogue no: SRL# 35657) and the protease inhibitor cocktail (Sigma Aldrich, Burlington, MA, USA, catalogue no:I3911-1BO) were donated by TAAB Biostudy Services. The aldosterone, C_21_H_28_O_5_ (Sigma Aldrich, Burlington, MA, USA, catalogue no: A9477), adrenaline tartrate, C_9_H_13_NO_3_·C_4_H_6_O_6_ (Sigma Aldrich, Burlington, MA, USA, catalogue no: A0300000), noradrenaline, C_8_H_11_NO_3_·C_4_H_6_O_6_·H_2_O (Sigma Aldrich, Burlington, MA, USA, catalogue no: N1100000) and the Atrial Natriuretic peptide, C_64_H_107_N_25_O_19_S_2_ (Sigma Aldrich, Burlington, MA, USA, catalogue no: SCP0022)were employed. 

### 2.2. Animals and Experimental Protocol

Male Wistar albino rats (8–9 weeks) weighing 130–150 g were purchased from Chakraborty Enterprises (West Bengal, India). All of the animal experiments were approved by the IAEC (1938/PO/Rc/S/17/CPCSEA).To rule out any intercurrent infection, they were kept under observation for roughly ten days prior to the start of the experiment. The animals were kept in polypropylene cages with well-aerated stainless steel covers and were exposed to a regular 12-h light/dark cycle and ambient temperature.

### 2.3. Development of Hypertensive Model in Rats

Hypertension was artificially induced in the Wistar rats by intraperitoneal administration of L-NAME (N-nitro-l-arginine methyl ester) in a dose of 185 mmol/kg of body mass two times daily for 7 consecutive days [8].

### 2.4. Non-Invasive Blood-Pressure Measurement 

Using the BIOPAC-MP36 system, the systolic blood pressure (SBP) was recorded non-invasively by inserting a cuff around the tails of the adult male rats (kept in approved animal restrainers) (BIOPAC System Inc., Goleta, CA, USA). The test-animals’ body temperatures were artificially maintained at 37 °C using an animal-heating unit. A non-invasive blood-pressure device (NIBP 200A, Santa Barbara, CA, USA) for animal tail insertion and finally a data simulation device (MP36, Goleta, CA, USA) were employed to acquire the data. The blood pressure was recorded until consistent and repeatable results were achieved [8].

### 2.5. Surface Electrocardiogram (ECG) Measurement in Anesthetized Rats

Ketamine (60 mg·kg^−1^) and xylazine (10 mg·kg^−1^) were used to anesthetize the rats, as reported previously. Using a standard lead, an ECG was taken for 5 min, immediately after anesthesia. MP36 (BIOPAC, Goleta, CA, USA) was used to collect and analyze the ECG signals. The RR-interval was measured on day 0 (self-control) i.e., before the L-NAME administration, day-7 (L-NAME) and day-14 (L-NAME +respective formulations) [9].

### 2.6. Allocation of Groups: Animals Were Divided into Six Groups (n = 6)

**Group I or hypertensive control group:** L-NAME administered for 7 days and left untreated for the next 7 days

**Group II or KASgroup:** L-NAME administered (i.p) for 7 days and L-NAME + KAS(1000 mg/kg b.w) for the next 7 days.

**Group III or DMM group:** L-NAME administered (i.p) for 7 days and L-NAME + DMM(2000 mg/kg b.w) for the next 7 days.

**Group IV or MSR group:** L-NAME administered (i.p) for 7 days and L-NAME + MSR(300 mg mg/kg b.w) for the next 7 days.

**Group V or HJ group:** L-NAME administered (i.p) for 7 days and L-NAME + HJ(113 mg/kg b.w) for the next 7 days.

**Group VI or KGS group:** L-NAME administered (i.p) for 7 days and L-NAME +KGS (2000 mg/kg b.w) for the next 7 days.

The animals were divided into six groups (*n* = 6) and the entire protocol comprised of 14 days. The control group was treated with L-NAME for 7 consecutive days and left untreated for the next 7 days. The animals of Group II-VI were initially treated with L-NAME for the first 7 days and concurrently administered with the respective formulations for the next 7 days. The RR-interval and systolic blood pressure (SBP) were measured on the 0th, 7th and 14th day. All of the unani formulations were administered orally using water as the vehicle.


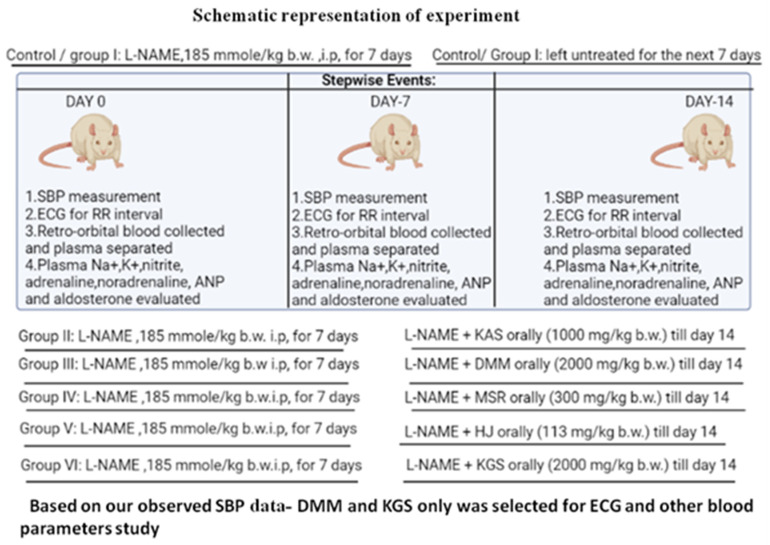


### 2.7. Preparation of 0.2M Phosphate Buffer Solution pH 7.5

A total of 27.22 gm of potassium dihydrogen phosphate was dissolved in 930 mL of water, the pH was adjusted to 7.5 using 300 gm/L solution of potassium hydroxide and finally, the volume was made upto 1000 mL with water [10].

### 2.8. Collection of Plasma for Quantification of Biomarkers

Less than 1 mL of the blood samples from the retro-orbital plexus was collected and separated by centrifugation for 10 min at 3500 rpm in a cold centrifuge (4 °C). The plasma was collected separately in two parts. The first part was analyzed for sodium [11,12], potassium by Elyte 3 kit [13] and nitrite by Griess Assay [14] for the respective groups while the second part contained the protease inhibitor cocktail for the adrenaline, noradrenaline, ANP and aldosterone estimation by LC-MS/MS. The protease-inhibitor cocktail powdered product was dissolved by adding 1ml of deionized water into the bottle to make a 100 times concentrated-stock solution. The buffer was diluted to make 100 mL of 1× (1:100) protease inhibitor solution. A total of 10 µL of the protease inhibitor solution was added to 2 mL of the plasma sample and immediately mixed [15]. The samples were stored at −20 °C.

### 2.9. LC-MS/MS Methodology

To acquire data with a very low limit of quantification, which is essential for recording extremely minute changes in the blood concentration of hormones and neurotransmitters following the administration of test substances, is only possible with a LC-MS/MS triple-quadruple quantification. Shimadzu LC was used with a binary pump, on-line degasser, and a thermostatic autosampler (SIL-20AC). The LC was coupled to an Ab Sciex API-4000 QTRAP mass spectrometer equipped with an electrospray ionization (ESI) source. The Analyst Software 1.6.3 was used for the data acquisition. The separation was achieved using Phenomenex Kinetex (5µ C18 100A 50 × 3 mm)

#### Chromatographic Conditions

The injection volume was 10 μL. The mobile phases A and B were eluted in a linear gradient at a flow rate of 0.5 mL/min, while the column was kept at a constant temperature of 25 °C. The ionization source was electrospray ionization (ESI) in the positive and negative ion modes. Nitrogen was used as the curtain gas and was maintained at a flow of 20.00 psig with GS1 and GS2 gas at 40.00 and 45.00 psig, respectively. The gas temperature was set at 400 °C and the capillary voltage was −4500.00 V. The declustering potential was applied for parent ionization and the collision energy for the product ionization. Multiple reaction monitoring was the final stage of the detector response of the mass spectrometry. ESI was taken as the ion source. adrenaline, noradrenaline, aldosterone and atrial natriuretic peptide were extracted from the plasma matrix, using solid phase extraction (solid phase extraction cartridge). For the assessment of the aldosterone, tolbutamide was taken as the internal standard (IS), while propranolol served as an internal standard (IS) for ANP, adrenaline and noradrenaline.

## 3. Statistical Analysis

An average of three readings were taken over a period of 2 h and the variation was observed in the second place of the decimal. The mean ± standard deviation was used to represent all of the data. GraphPad Prism 9.0 was used to undertake the statistical data analysis (Graph Pad, San Diego, CA, USA). One-way ANOVA was applied to assess all of the serum parameters, and Tukey’s test was used for post-hoc analysis. A statistically significant value was defined as *p* < 0.05.

## 4. Results

**Figure 1:**
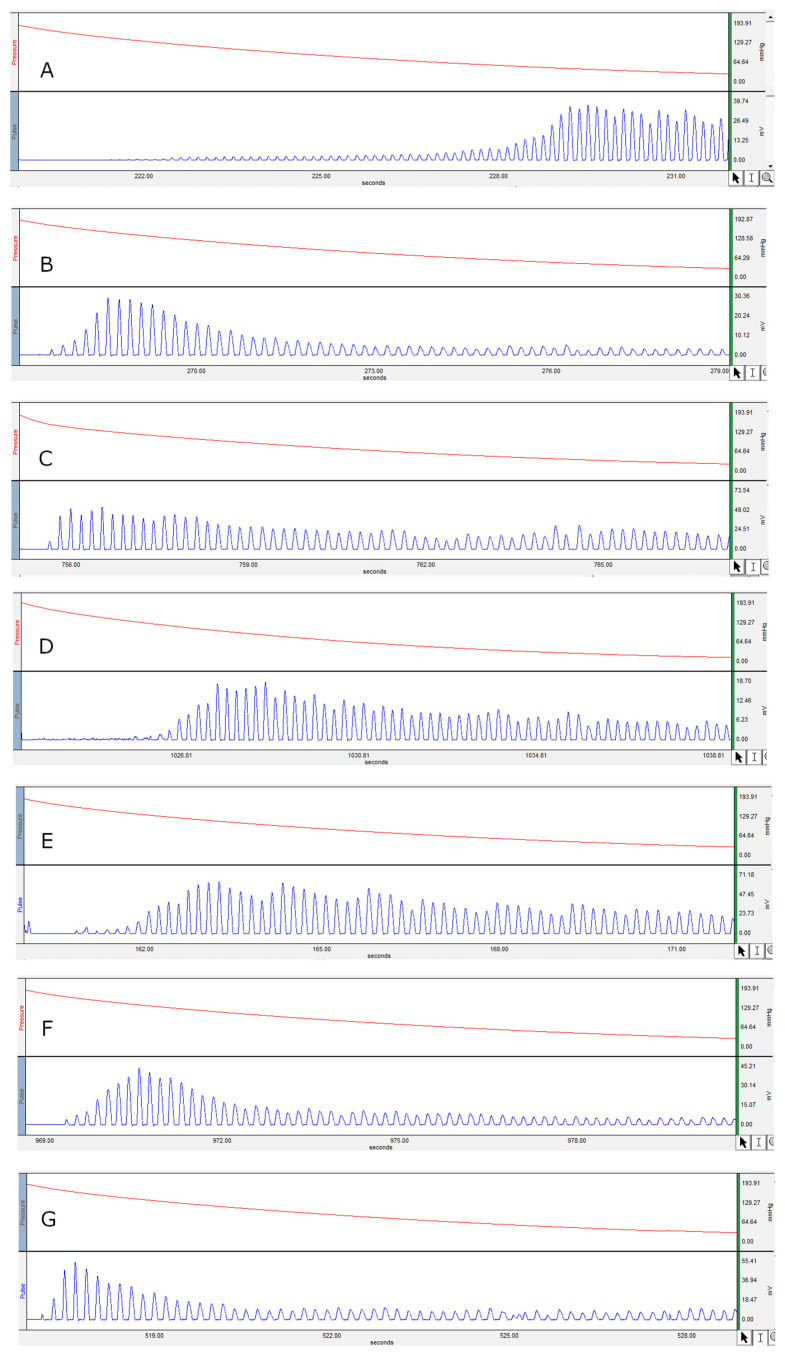
Represents the non-invasive systolic blood pressure. (**A**) Hypertensive control on day 0; (**B**) SBP on day 7 on treatment with L-NAME; (**C**) SBP of KAS on day 14; (**D**) SBP of DMM on day 14; (**E**) SBP of MSR on day 14; (**F**) SBP of HJ on day 14; (**G**) SBP of KGS on day 14.

**Figure 2:**
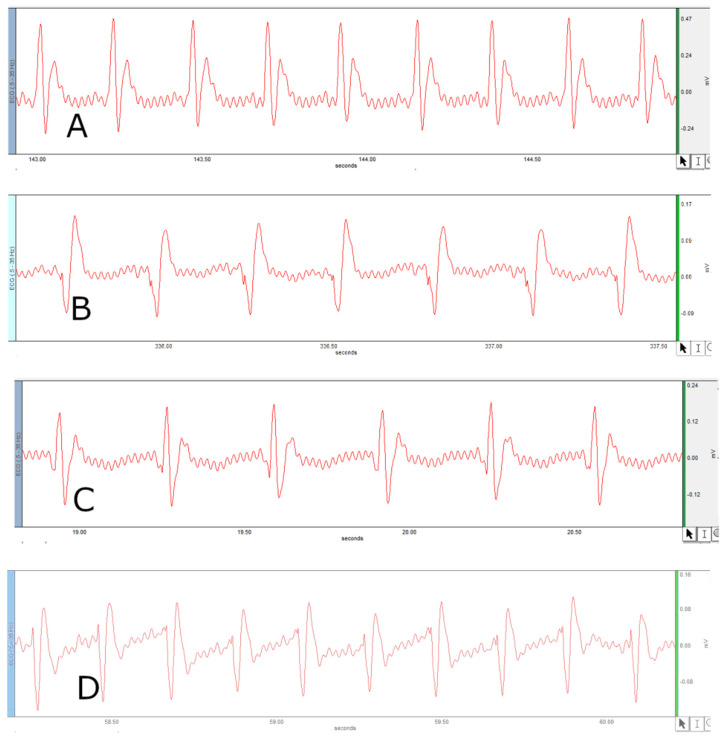
Represents RR-interval. (**A**) RR-interval of control on Day 0; (**B**) RR-interval on day 7 on treatment with L-NAME; (**C**) RR-interval of KGS on day 14; (**D**) RR-interval of DMM on day 14.

**Figure 3:**
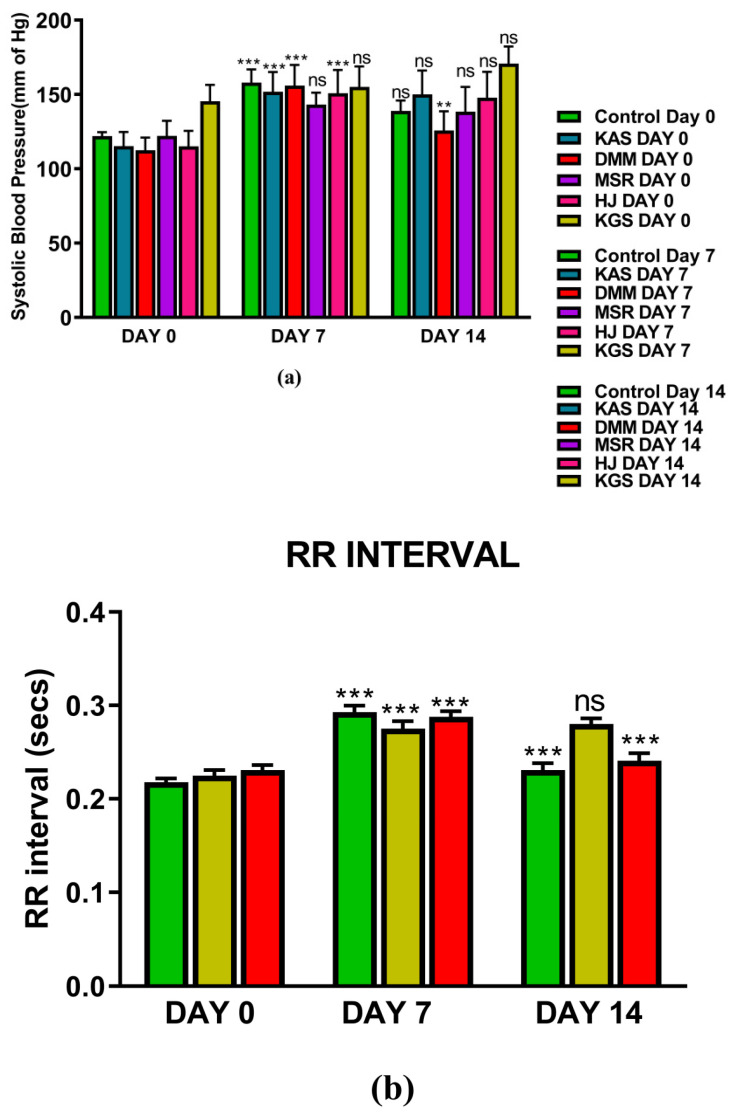
(**a**) Represents mean ± SD values of non-invasive systolic blood pressure of hypertensive control, KAS, DMM, MSR, HJ and KGS on day 0,7 and 14; (**b**) Represents RR-interval of DMM and KGS on day 0, 7 and 14. ** *p* < 0.01, *** *p* < 0.001, ns-non-significant. Day 0 vs. Day 7, and Day 7 vs. Day 14.

**Figure 4:**
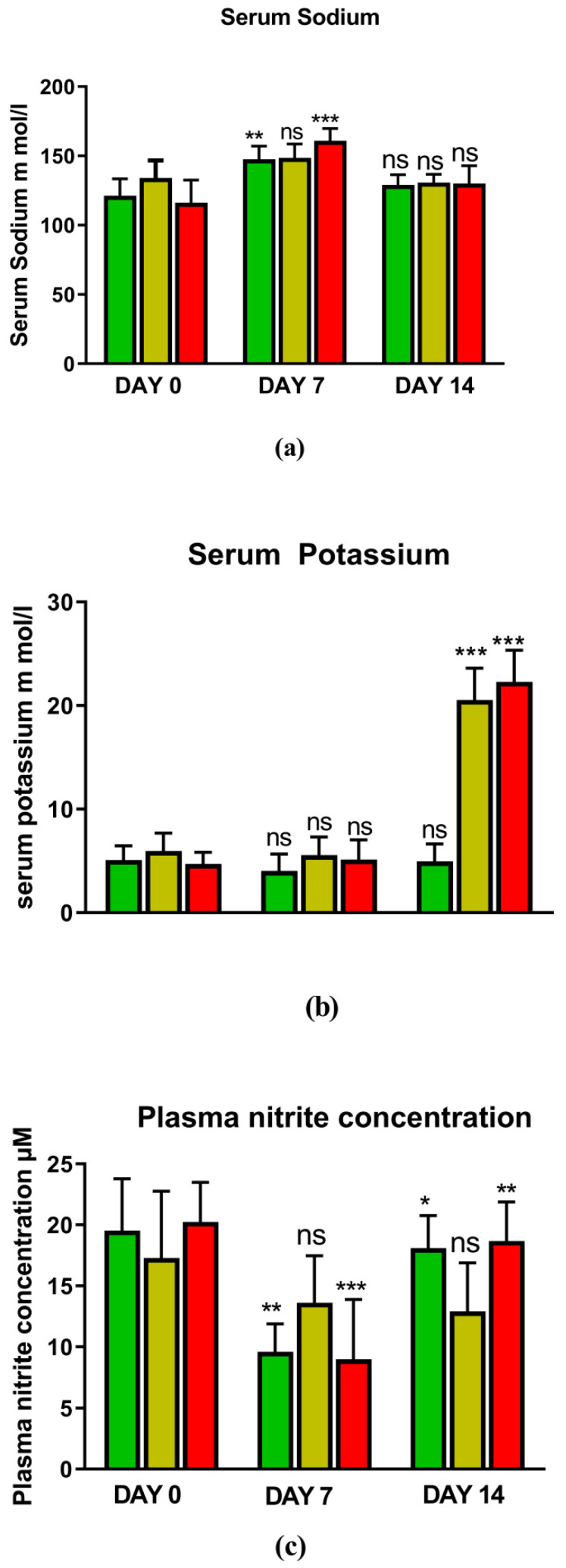
Represents (**a**) Plasma sodium; (**b**) Plasma potassium; (**c**) Plasma nitrite of control, KGS and DMM on day 0, 7 and 14. * *p* < 0.05, ** *p* < 0.01, *** *p* < 0.001, ns-non-significant. Day 0 vs. Day 7, and Day 7 vs. Day 14.

**Figure 5:**
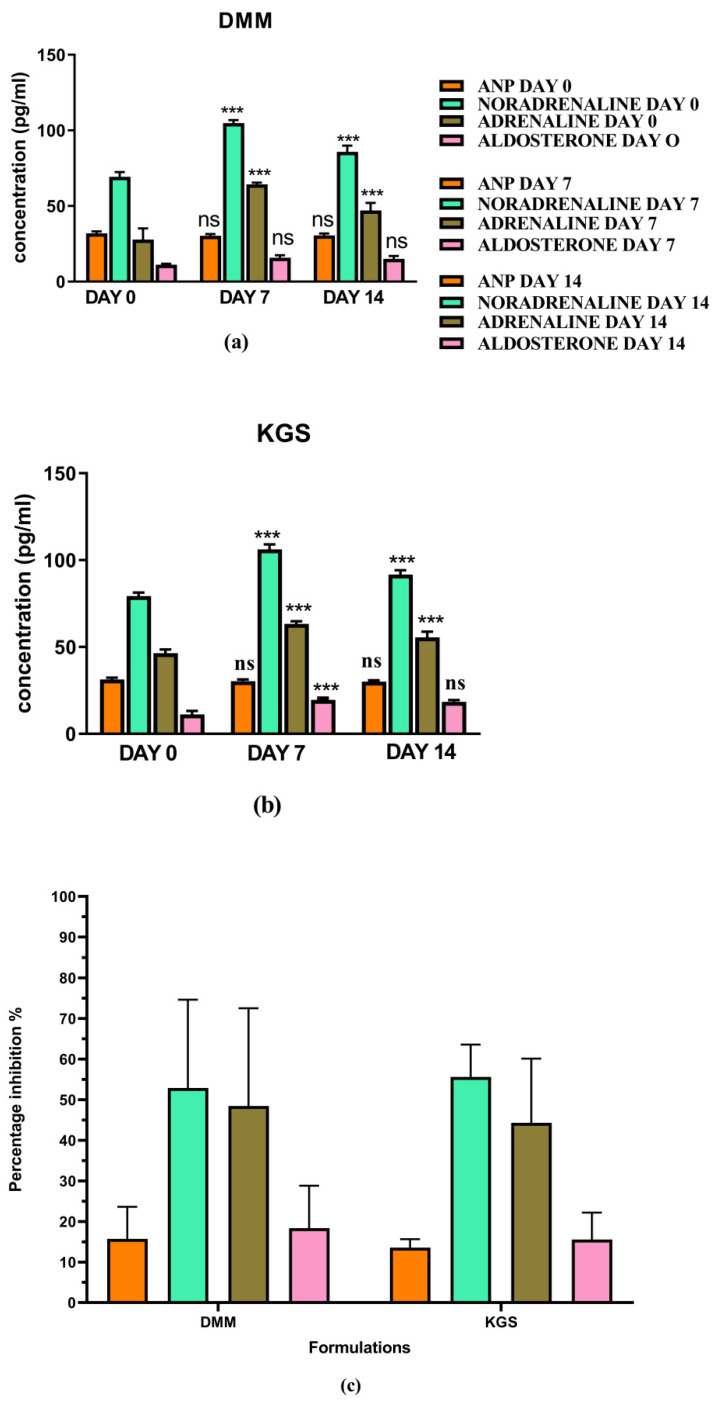
(**a**,**b**) Plasma concentration of ANP, noradrenaline, adrenaline and aldosterone on day 0, 7 and 14 in DMM- and KGS-treated rats; (**c**) % inhibition on day 14 as compared to day 7 by KGS and DMM. ns—non-significant, *** *p* < 0.001. Day 0 vs. Day 7, and Day 7 vs. Day 14.

**Figure 6:**
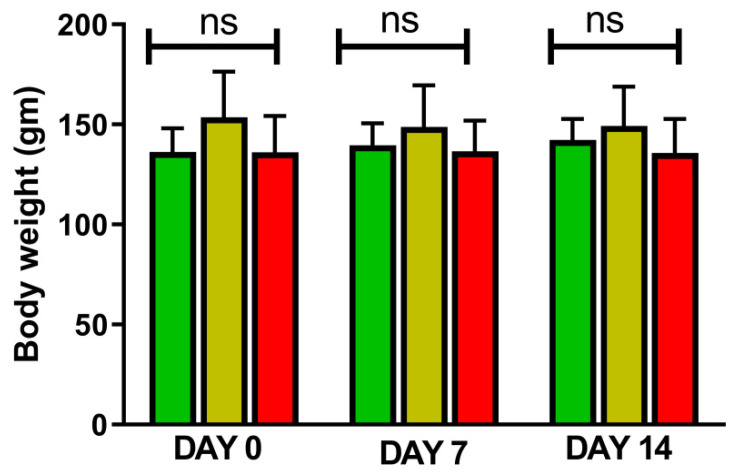
Represents mean ± SD of Body weight of rats on day 0,7 and 14. ns—non-significant. Day 0 vs. Day 7, and Day 7 vs. Day 14.

**Figure 7:**
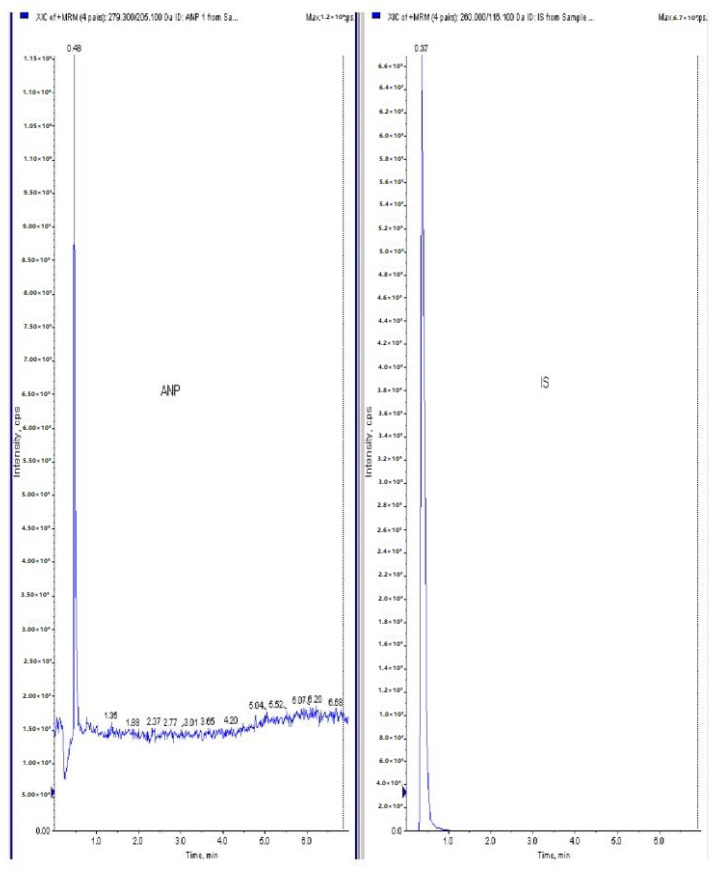
Raw data peaks obtained from LC-MS/MS quantitative measurements (MRM) of ANP using propranolol as internal standard (IS).

**Figure 8:**
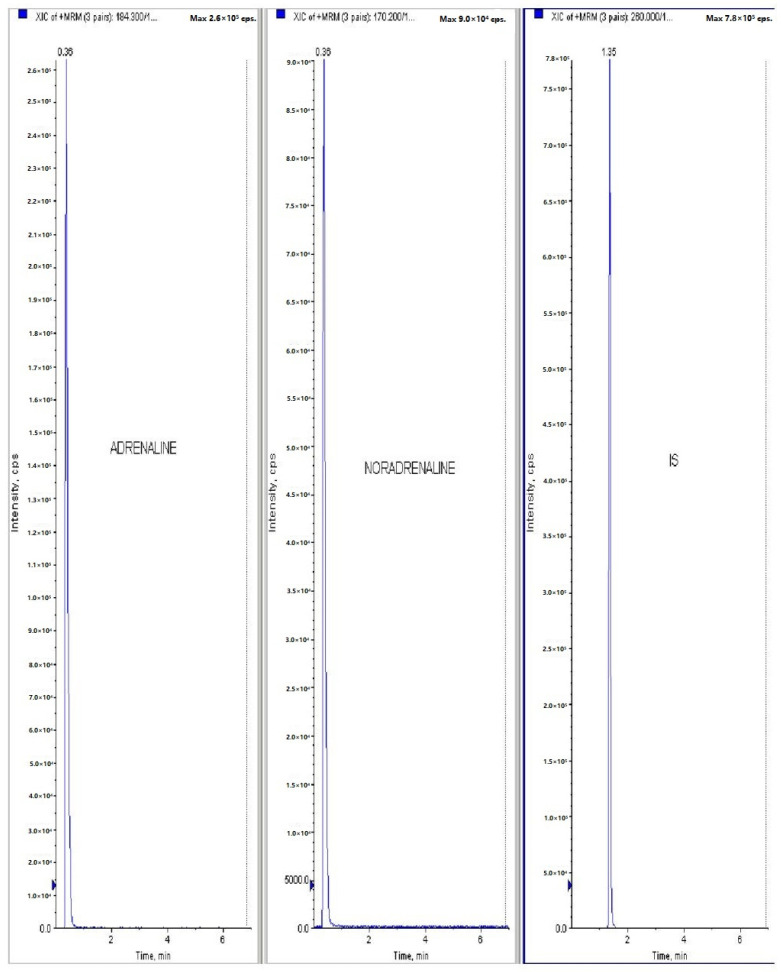
Raw data peaks obtained from LC-MS/MS quantitative measurements(MRM) of adrenaline and noradrenaline using propranolol as internal standard (IS).

**Figure 9:**
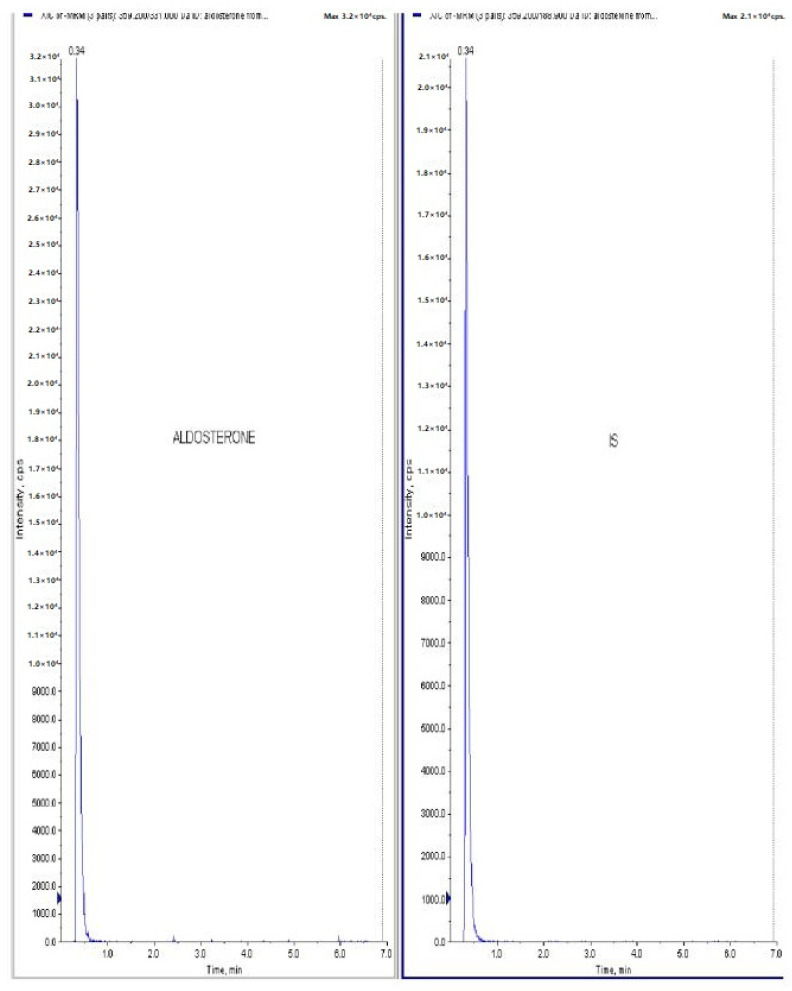
Raw data peaks obtained from LC-MS/MS quantitative measurements (MRM) of aldosterone using tolbutamide as internal standard (IS).

Figure 1 and Figure 2 represents the non-invasive SBP and RR-interval. Figure 3a shows non-significant decrease in KAS, MSR and HJ on day 14, as compared to day 7, but a significant (*p* < 0.05) one was noted in DMM. Unexpectedly, an elevated SBP was found in the KGS-treated group on day 14. There was a significant lengthening of the RR-interval on day 7. The shortening of the RR-interval on day 14 was not seen in KGS, on the contrary it lengthened, while the control and the DMM group showed a significant decrease (*p* < 0.05) (Figure 3b). The increase in the serum sodium for the control, KGS and DMM groups on day 7 were significant, except for the KGS group; meanwhile, on day 14, all of the groups showed a non-significant slight reduction (Figure 4a). The plasma potassium did not much change on day 7for all of the groups, while on day 14 it significantly (*p* < 0.05) increased, but not in the control group (Figure 4b). In Figure 4c, the plasma nitrite was found to decrease significantly (*p* < 0.05) in the control and DMM group on day 7 after the L-NAME treatment, but the animals in the KGS group showed a non-significant decrease on day 7. On day 14, there was not much change in the plasma nitrite status in the animals of the KGS group, while the control and DMM groups displayed a significant (*p* < 0.05) increase. In Figure 5a,b, the plasma adrenaline and noradrenaline in both the DMM- and KGS-treated rats increased significantly (*p* < 0.05) on day 7, and similarly the decrease on day 14 was also noted to be significant (*p* < 0.05). In Figure 5a,b there was no significant change in the plasma ANP and aldosterone in both the DMM and KGS groups for day 7 and 14. Figure 5c depicts the % inhibition in both the DMM and KGS group on day 14, in comparison to day 7. Figure 6, Figure 7, Figure 8 and Figure 9 represent the MRM (Multiple Reaction Monitoring) chromatogram of ANP, noradrenaline, adrenaline and aldosterone. The adrenaline, noradrenaline and ANP were ionized in the positive mode for detection, while aldosterone was evaluated by ionizing in the negative mode. Similarly, Figure 6 represents the body weight of animals and did not show much change (non-significantly) from day 0–14.

## 5. Discussion

Blood pressure is mainly modulated by cardiac output and total peripheral resistance (t.p.r). In this experiment, the systolic blood pressure of some of the selected unani formulations was measured against L-NAME-induced hypertension. L-NAME is a NOS (nitric oxide synthase)-inhibiting chemical moiety, which leads to NO (nitric oxide)-deficient artificial hypertension in experimental animals. However, the NOS distribution has been found in both the central and peripheral nervous system which modulates the cardiovascular functions through the autonomic system. NOS inhibition at the CNS level might also contribute to hypertension [16]. Apart from NO inhibition, other mechanisms through which L-NAME acts include SNS (sympathetic nervous system) activation, RAAS activation and oxidative stress. The adrenergic activation by L-NAME leads to the release of adrenaline and Noradrenaline, eventually exerting positive inotropic and chronotropic effect on the myocardium causing elevated blood pressure. It also acts by increasing Renin and Ang-II levels in the renal system, thereby augmenting plasma aldosterone and retaining Na^+^ and water [17].

The endothelium slowly releases a small amount of NO at baseline conditions, maintaining the constant vasodilation of the vascular smooth muscles [18]. The administration of L-NAME inhibits NOS, reducing the concentration of vasodilatory NO, causing vasoconstriction, eventually leading to an elevation in SBP. NO activates the soluble guanyl cyclase and this in turn causes the generation of cGMP. Nitric oxide oxidation leads to the production of nitrite found in the plasma [19]. It has been reported that bolus administration of L-NAME causes an increase in blood pressure with a concomitant decrease in heart rate or the lengthening of the RR-interval in ECG recordings [20]. The heart rate is reciprocally associated with the RR-interval (QRS-to-QRS interval) [21]. The presence of long RR-intervals may worsen CHD (coronary heart disease) and HF (heart failure), as well as elevating the SBP [22]. The recordings of the non-invasive SBP (Figure 1) and RR-interval (Figure 2) in this experiment on day 7 as compared to day 0 in all of the groups corroborate with the earlier reports of L-NAME administration [20].

### 5.1. Summary of Observation on Day 7

The SBP and RR-interval was found to increase significantly (*p* < 0.05) in all of the groups after 7 days of i.p. administration of L-NAME, except for the SBP of the MSR and KGS groups, which showed a non-significant increase.

#### 5.1.1. DMM

Both the plasma adrenaline and noradrenaline increased after the L-NAME treatment on day 7 in the DMM group, which was consistent with previous scientific reports. The plasma potassium level did not sufficiently rise on the 7th day for the DMM group.

#### 5.1.2. KGS

Both the plasma adrenaline and noradrenaline increased after the L-NAME treatment [17] on day 7 in the KGS group. The plasma potassium level did not sufficiently rise on the 7th day for the KGS group.

The concentration of noradrenaline was predominantly higher than adrenaline on day 7 in both of the groups, which might have contributed to the decreased HR or the lengthening of the RR- interval via β_1_ agonism. Through the dilation of the afferent arterioles and the constriction of the efferent arterioles, ANP increases the glomerular filtration rate (GFR) in the kidney. Further, at various regions of the nephron, ANP inhibits the salt and water reabsorption, while renin and the related plasma-aldosterone does the reverse [23]. Previous scientific reports suggest that the administration of L-NAME elevates plasma-ANP and induces its release through the enhancement of volume-load in conscious rats [24]. ANP is also released due to atrial stretch caused by the action of NA on α receptors or adrenaline on β receptors. On day 7, the L-NAME group showed a significant increase in NA, Adrenaline and SBP, while the ANP and aldosterone remained almost unaltered. A probable reason for this might be the insufficient stretching of the atrial walls to release ANP and a milder alteration of plasma Na^+^, as was evident in our Figure 4a, to trigger a significant increase in aldosterone. Unfortunately, we could not measure the atrial pressure or the parameters that give information about atrial stretch. It is interesting to mention that the literature also indicates that the plasma concentration of ANP is independent of the concentration of plasma adrenaline and noradrenaline [23].

### 5.2. Summary of Observations on Day 14

On day 14, the SBP of the animals treated with KAS, MSR and HJ showed a non-significant decrease. 

#### 5.2.1. DMM

The decrease in SBP was significant (*p* < 0.05) in the case of the DMM-treated rats. The RR-interval on day 14 significantly decreased in the DMM group. This change was conventional in the case of DMM, i.e., typical of an anti-hypertensive agent. The significant (*p* < 0.05) rise in the nitrite levels on day 14 indicate a different mechanism for DMM, exerting its vasodilatory effect by countering the L-NAME inhibition of NOS. The DMM also decreased plasma NA and adrenaline but showed an increased heart rate (shortening of the RR-interval) on day 14 (Figure 3b and Figure 5a). This can be explained by relating that an increase in the plasma catecholamines downregulates the adrenergic receptors, leading to diminished activity, according to some scientific reports [25]. It is probably that NA fails to achieve pharmacological action because of the higher concentration and the activity of adrenaline overshadows that of noradrenaline, leading to elevated HRs, a possible explanation for the increased HR produced by DMM on day 14 (Figure 3b). The plasma potassium levels were significantly elevated on the 14th day in the DMM-treated rats. Reports suggest that an increase in extracellular potassium is linked to the depolarization of smooth muscle cells, causing vasodilation [26]. The decrease in SBP and elevation of K^+^ on the 14th days can be extrapolated to our findings. However, the significant (*p* < 0.05) rise in the nitrite levels on day 14 (Figure 4c) indicate a different mechanism for the DMM, exerting its vasodilatory effect by countering the L-NAME inhibition of NOS. The DMM also decreased the plasma NA and adrenaline but showed increased heart rates (shortening of the RR-interval) on day 14 (Figure 5a). This can be explained by relating that an increased plasma catecholamine level downregulates the adrenergic receptors, leading to diminished activity, according to some scientific reports [25]. It is probably that NA fails to achieve pharmacological action because of the higher concentration, and because the activity of adrenaline overshadows that of noradrenaline, leading to elevated HRs, a possible explanation for an increased HR (shortening of the RR-interval) produced by DMM on day 14 (Figure 3b).

#### 5.2.2. KGS

Unexpectedly, the KGS showed an enhancement in the SBP and RR-interval, in the KGS group, rather than decreasing on day 14, though not significantly. It is unclear why the RR and SBP increased non-significantly in the case of KGS. The observed decrease in the plasma concentration of adrenaline and noradrenaline from day 7 to day 14, following administration of the KGS appears to be similar to that of α_2_ agonist e.g., clonidine [27]. Strangely enough, the plasma nitrite did not increase on day 14, instead it decreased a bit in the case of KGS, which in another way supports the enhanced SBP in this group (Figure 4c). On the treatment of rats with KGS, on day 14, the noradrenaline and adrenaline decreased yet the concentration of noradrenaline was predominantly higher than adrenaline, which might explain a lower heart rate (increased RR-interval) (Figure 3b). Thus, it might be said that the KGS might not alter cardiac output (pharmacological action of noradrenaline) but may increase stroke volume and decrease the HR, indicating that it might exert a central sympathomimetic role. We do not have many reports to justify an elevated SBP and K^+^ in the KGS-treated rats on day 14 (Figure 3a and Figure 4b). So far, the convention is concerned that high plasma K^+^ indicates a smaller RR-interval which is expected to generate lower systolic blood pressure. In our study, we observed the reverse co-relationship between the RR-interval and K^+^. Therefore, it will be pertinent to mention in this context, one study claims an association of high plasma K^+^ accompanied by high blood pressure [28].

The plasma aldosterone did not change much from day 7 to day 14 in both the KGS- and DMM-treated rats. This is also quite evident from the non-significant slight decrease in Na^+^ level on day 14; such a small change may not be attributed to aldosterone. Thus, the KGS and DMM might not exploit the ANP and aldosterone balance and ultimately may not influence the Angiotensin-II release.

## 6. Conclusions

In our experiment, it is quite interesting to mention that KAS, MSR and HJ were unable to reduce the L-NAME-induced blood pressure, while DMM reduced the SBP. Perhaps, DMM acts through the inhibition of NOS as well as the adrenergic system. Our findings have also revealed a strange observation in the context of the way KGS acts, especially in elevating the SBP and K^+^ and slightly increasing the RR-interval. However, we have put forward a single previous report from the *American Journal of Hypertension* [28] which might help to explain, at least in part, the observation in relation to KGS mentioned earlier. KGS has shown some of the remarkable pharmacological features similar to that of clonidine. This finding indicates that probably KGS acts as a central sympathomimetic agent. Thus, more detailed studies of KGS are needed to justify that the observed increase in SBP here maybe clinically correlated with its traditional use.

## Figures and Tables

**Table 1:** Composition of Khamira Gozaban Sada (KGS) as per label claim.

SL.No	Composition	Part Used	Quantity
1	Abresham muqarraz	-	21.93 mg
2	Badranjboya (*Nepeta hindostana* (B.Heyne ex Roth) Haines)	Flower	153.51 mg
3	Berg e Gawzaban (*Borago officinalis* L.)	Leaf	131.58 mg
4	Burada Sandal Safaid (*Santalum album* L.)	Heart wood	109.65 mg
5	Behman Surkh (*Salvia haematodes* L.)	Root	87.72
6	Tukhm Balangu (*Lallemantia royleana* (Benth)Benth.	Seed	109.65 mg
7	Tudri Surkh (*Cheiranthus cheiri* L.)	Seed	43.86 mg
8	Kishneez Khushk (*Coriandrum sativum* L.)	Fruit	131.58 mg
9	Gule Khatmi (*Althaea officinalis* L.)	Flower	43.86 mg
10	Gule Gawzaban (*Borago officinalis* L.)	Flower	43.86 mg
11	Shakar safaid (*Saccharum officinarum*)	Crystal	8.722 g
12	Sat Leemun (*Citrus aurantium* L.)	Crystal	17.54 mg
13	Natroon Banjawi	-	5.26 mg

**Table 2:** Composition of Dawa ul Misk Motadil (DMM) as per label claim.

SL.No	Composition	Part Used	Quantity
1	Zarishk (*Berberis aristata* DC)	Fruit	132.0 mg
2	Tabasheer Safaid (*Bambusa arundinace* willd.)	Silicacious concretion	88 mg
3	Sandal safaid (*Santalum album* L.)	Heart wood	88 mg
4	Sandal Surkh (*Pterocarpus santalinus* L.f.)	Heart wood	88 mg
5	Kishneez Muqashshar (*Coriandrum sativum* L.)	Dried seed	88 mg
6	Gule Gawzaban (*Borago officinalis* L.)	Flower	88 mg
7	Amla (*Embelica officinalis*)	Dried fruit	88 mg
8	Tukhm Khurfa (*Portulaca oleracea* L.)	Dried seed	88 mg
9	Gule Surkh (*Rosa damascene* Herrm.)	Flower	51 mg
10	Abresham Muqarraz (*Bombyx mori* cocoon)	Cocoon	51 mg
11	Darchini (*Cinnamomum zeylanicum* Blume)	Stem, Bark	51 mg
12	Behman Safaid (*Centaurea behen* L.)	Root	51 mg
13	Behan Surkh (*Salvia haematodes* L.)	Root	51 mg
14	Darunaj Aqrabi (*Doronicum hookeri* C.B.Clarke ex Hook.f)	Rhizome	51 mg
15	Ood Hindi (*Aquilaria agallocha* Roxb.)	Heart wood	36 mg
16	Badranjboya (*Nepeta hindostana* (B.Heyne ex Roth) Haines)	Whole plant	36 mg
17	Mastagi (*Pistacia lentiscus* L.)		30 mg
18	Ushana (*Usnea longissima* Ach.)	Thallus	30 mg
19	Dana Elaichi Khurd (*Elettaria cardamomum* L.)	Dried seed	30 mg
20	Qand Safaid (*Saccharum officinarum*)	Crystal	2.44 g
21	Shahad (Honey)	--	1.22 g
22	Aabe Seb Shinn (*Pyrus malus* L.)	Fruit	1.22 g
23	Zafran (*Crocus sativa* L.)	Stigma	51 mg
24	Amber (resin) without musk	-	14 mg

**Table 3:** Composition of Khamira Abresham Sada as per label claim.

SL.No	Composition	Part Used	Quantity
1	Abresham Muqarraz	Cocoon	603.3 mg
2	Berg Badranjboys ((*Nepeta hindostana* (B.Heyne ex Roth)) Haines)	Leaf	452.4 mg
3	Berg Gawzaban (*Borago officinalis* L.)	Leaf	262 mg
4	Gule Khatmi (*Althaea officinalis* L.)	Flower	111.6 mg
5	Shakar Safaid (*Saccharum officinarum*)	Crystal	9.049 g
6	Sat Leemun (*Citrus aurantium* L.)	Crystal	36.20 mg
7	Natroon Banjawi		9.049 mg
8	Zafran (*Crocus sativus* L.)	Stigma	5.279 mg
9			

**Table 4:** Composition of Habb-e-Jadwar as per label claim.

SL.No	Composition	Part of the Plant	Quantity
1	Afyun (*Papaver somniferum* L.)	Fruit	25.68 mg
2	Jadwar (*Delphinium denudatum* Wall. ex Hook.f. and Thomson)	*Tuber*	5.136 mg
3	Zafran *(Crocus sativa* L.)	*Style and Stigma*	2.568 mg
4	Sheer Gao *(Cow Milk)*		6.420 mL
5	Narjeel *(Cocos nucifera* L.)	*Kernel*	102.72 mg
6	Bisbasa *(Myristica fragrans* Houtt)	*fruit coat*	
7	Behman safed (*Centaurea behen* L.)	*Root*	6.629 mg
8	Behman surkh *(Salvia heamatodes* L.)	*Root*	6.629 mg
9	Maghz Badam Shirin *(Prunus amygdalus L.)*	*Stem oil*	9.946 mg
10	Maghz Chilghoza *(Pinus gerardiana* Wall.)	*Kernel*	9.946 mg
11	Tukhme Khurfa *(Portulaca oleracea* L.)	*Seed*	9.946 mg
12	Roghan Balsan *(Commiphora opobalsamum* L.)	*Oil and Stem*	13.268 mg
13	Jawitri (*Myristica fragrans* Houtt.)	*Aril*	6.629 mg
14	Badranjboya (*Nepeta hindostana* (B.Heyne ex Roth) Haines)	*Whole plant*	6.629 mg
15	Banslochan (*Bambusa arundinacea* willd.)	Silicacious concretion	2.94 mg
16	Gond Keekar (*Acacia arabica*)	*Gum*	2.94
17	Misri (*Saccharum officinarum*)	*Cubes*	13.268 mg
18	Kaleera (*Cochlospermum religiosum* (L.) Alston)	Gum	2.94 mg
19	Ajwain Khurasani (*Hyoscyamus niger* L.)	Fruit	2.94
20	Beikh Luffah *(Atropa Belladona* L.)	Root	2.94
21	Jaiphal (*Myristica fragrans* Houtt.)	Seed	2.94
22	Warq Nuqra (silver)		7.210 mg
23	Ghee		q.s.

**Table 5:** Composition of Mufarreh Shaikhur Raees as per label claim.

SL.No	Composition	Part of the Plant	Quantity
1	Agar (*Aquilaria agallocha* Roxb.)	Heart wood	37 mg
2	Elaichi Khurd (*Elettaria cardamomum* L.)	Fruit	55.5 mg
3	Berg Gawzaban (*Borago officinalis* L.)	Leaf	92.5 mg.
4	Burada Sandal Surkh (*Pterocarpus santalinus* L.f.)	HeartWood	27.75 mg
5	Burada Sandal Safaid (*Santalum album* L.)	HeartWood	55.5 mg
6	Banslochan (*Bambusa arundinacea* willd)	Silicaceous concretion	55.5 mg
7	Behman Surkh (*Salvia haematodes* L.)	Root	37 mg
8	Tukhm Khurfa Siyah (*Portulaca oleracea* L.)	Seed	83.25 mg
9	Tukhm Kahu (*Lactuca sativa* L.)	Seed	83.25 mg
10	Qust Shireen (*Saussurea lappa* (Falc.) Lipsch.)	Root	37 mg
11	Zarambad (*Curcuma zedoaria* Roxb.)	Root Trunk	37 mg
12	Sartan Sokhta (*Scylla serrata* (Forskål, 1775) ash)	-	27.75 mg
13	Gule Surkh (*Rosa damascene* Herrm.)	Flower	138.75 mg
14	Maghz Tukhm Kharbooza *(Cucumis melo* L.)	-	83.25 mg
15	Maghz Tukhm Khayarain *(Cucumis sativus* L.)	-	83.25 mg
16	Maghz Kadu Shireen (*Cucurbita maxima* Duch ex.Lam)	-	83.25 mg
17	Kafoor (Camphor)	-	27.75 mg
18	Busad Sokhta Mehlool	-	27.75 mg,
19	Marwareed Mehlool	-	27.75 mg
20	Abresham Muqarraz	Cocoon	27.75 mg
21	Qwam Shakar Safaid (*Saccharum officinarum*)	Crystal	2.222 g
22	Rub Anar Shireen (*Punica granatum* L.)	Fruit	555 mg
23	Rub Behi (*Cydonia oblonga* Mill)	Fruit	555 mg
24	Rub Seb (*Malus sylvestris* (L.) Mill.)	Fruit	555 mg
25	Natroon Banjawi.	-	5.55 mg
26	Zafran (*Crocus sativa* L.)	Stigma	6.48 mg
27	Arq Gawzaban (*Borago officinalis* L.)	Distilled	0.07 mL
28	Warq Nuqra	-	9.25 mg

## Data Availability

The data presented in this study are available on request from the corresponding author. The data are not publicly available as the machines are under the custody of the corresponding author.

## Abbreviations

CVD, cardiovascular disease; WHO, World Health Organization; L-NAME, N^ω^-nitro-L-arginine methyl ester; i.p., intraperitoneal; KAS, Khamira Abreesham Sada; DMM, Dawaul Misk Motadil; MSR, Mufarreh Shaikhur Rais; KGS, Khamira Gaozaban Sada, HS, Habb-e-Jadwar; ECG, electrocardiogram; SBP, systolic blood pressure; ANP, atrial natriuretic peptide; NCD, non-communicable disease; LC, liquid chromatography; QTRAP, quadruple ion trap; ESI, electrospray ionization; IS, internal standard; MRM, multiple reaction monitoring; tpr, total peripheral resistance; NOS, nitric oxide synthase; NO, nitric oxide; CNS, central nervous system; SNS, sympathetic nervous system; RAAS, renin-angiotensin-aldosterone system; Ang-II, angiotensin-II; cGMP, guanosine-3′,5′-cyclic monophosphate; CHD, coronary heart disease; HF, heart failure; HR, heart rate; GFR, glomerular filtration rate; NA, noradrenaline; Mfg, manufacturing; Lic, Licence.

## References

[1] Zhou B., Perel P., Mensah G.A., Ezzati M. (2021). Global epidemiology, health burden and effective interventions for elevated blood pressure and hypertension. Nat. Rev. Cardiol..

[2] Zhou X., Seto S.W., Chang D., Kiat H., Razmovski-Naumovski V., Chan K., Bensoussan A. (2016). Synergistic Effects of Chinese Herbal Medicine: A Comprehensive Review of Methodology and Current Research. Front. Pharmacol..

[3] Yuan H., Ma Q., Ye L., Piao G. (2016). The Traditional Medicine and Modern Medicine from Natural Products. Molecules.

[4] Ahmad S., Rehman S., Ahmad A.M., Siddiqui K.M., Shaukat S., Khan M.S., Kamal Y.T., Jahangir T. (2010). Khamiras, a natural cardiac tonic: An overview. J. Pharm. Bioallied Sci..

[5] Ministry of Health and Family Affairs (2006). Part V. National Formulary of Unani Medicine (NFUM).

[6] Ministry of Health and Family Affairs (2006). Part I. National Formulary of Unani Medicine (NFUM).

[7] Ravishankar B., Shukla V.J. (2007). Indian systems of medicine: A brief profile. Afr. J. Tradit. Complement. Altern. Med..

[8] Gorain B., Choudhury H., Kundu A., Sarkar L., Karmakar S., Jaisankar P., Pal T.K. (2014). Nanoemulsion strategy for olmesartan medoxomil improves oral absorption and extended antihypertensive activity in hypertensive rats. Colloids Surf. B Biointerfaces.

[9] Karmakar S., Padman A., Mane N.S., Sen T. (2013). Hypokalemia: A potent risk for QTc prolongation in clarithromycin treated rats. Eur. J. Pharmacol..

[10] Council of Europe, European Pharmacopoeia Commission, European Directorate for the Quality of Medicines & Healthcare (2010). European Pharmacopoeia.

[11] Maruna R.F. (1957). Serum sodium determination; critical study on colorimetric determination and method. Clin. Chim. Acta.

[12] Trinder P. (1951). Determination of serum sodium. Analyst.

[13] Suderman H.J., Delory G.E. (1952). A rapid method for the determination of sodium in serum. Can. J. Med. Sci..

[14] Lee A.Y., Choi J.W., Yokozawa T., Cho E.J. (2019). Preventive effect of oligonol on nitric oxide and reactive oxygen species production through regulation of nuclear factor kappa B signaling pathway in RAW 264.7 macrophage cells against sodium nitroprusside. RSC Adv..

[15] Shishkova E., Coon J.J. (2021). Rapid preparation of human blood plasma for bottom-up proteomics analysis. STAR Protoc..

[16] Pechanova O., Vrankova S., Cebova M. (2020). Chronic L-Name-Treatment Produces Hypertension by Different Mechanisms in Peripheral Tissues and Brain: Role of Central eNOS. Pathophysiology.

[17] Kvetnanský R., Pacák K., Tokarev D., Jeloková J., Jezová D., Rusnák M. (1997). Chronic blockade of nitric oxide synthesis elevates plasma levels of catecholamines and their metabolites at rest and during stress in rats. Neurochem. Res..

[18] Hildebrand S., Ibrahim M., Schlitzer A., Maegdefessel L., Röll W., Pfeifer A. (2022). PDGF regulates guanylate cyclase expression and cGMP signaling in vascular smooth muscle. Commun. Biol..

[19] Chowdhary S., Ng G.A., Nuttall S.L., Coote J.H., Ross H.F., Townend J.N. (2002). Nitric oxide and cardiac parasympathetic control in human heart failure. Clin. Sci..

[20] Sung J.H., Jo Y.S., Kim S.J., Ryu J.S., Kim M.C., Ko H.J., Sim S.S. (2013). Effect of Lutein on L-NAME-Induced Hypertensive Rats. Korean J. Physiol. Pharmacol..

[21] Stauss H.M. (2014). Heart rate variability: Just a surrogate for mean heart rate?. Hypertension.

[22] Xu H., Li J., Zhong G., Li L., Huang C., Guo P., Chen Y., He T. (2021). Characteristics of the Dynamic Electrocardiogram in the Elderly with Nonvalvular Atrial Fibrillation Combined with Long R-R Intervals. Evid. Based Complement. Alternat. Med..

[23] Ganguly A. (1992). Atrial natriuretic peptide-induced inhibition of aldosterone secretion: A quest for mediator(s). Am. J. Physiol..

[24] Leskinen H., Vuolteenaho O., Leppäluoto J., Ruskoaho H. (1995). Role of nitric oxide on cardiac hormone secretion: Effect of NG-nitro-L-arginine methyl ester on atrial natriuretic peptide and brain natriuretic peptide release. Endocrinology.

[25] Kotchen T.A., Tinsley R.H., Anthony S.F. (1998). Hypertensive vascular disease. Harrison’s Principles of Internal Medicine.

[26] Martinez D.V., Rocha R., Matsumura M., Oestreicher E., Ochoa-Maya M., Roubsanthisuk W., Williams G.H., Adler G.K. (2002). Cardiac damage prevention by eplerenone: Comparison with low sodium diet or potassium loading. Hypertension.

[27] Tripathi K.D. (2013). Essentials of Medical Pharmacology.

[28] Walsh C.R., Larson M.G., Vasan R.S., Levy D. (2002). Serum potassium is not associated with blood pressure tracking in the Framingham Heart Study. Am. J. Hypertens..

